# Phenotype and *in vitro *function of mature MDDC generated from cryopreserved PBMC of cancer patients are equivalent to those from healthy donors

**DOI:** 10.1186/1476-8518-5-7

**Published:** 2007-05-03

**Authors:** Smita A Ghanekar, Sonny Bhatia, Joyce J Ruitenberg, Corazon DeLa Rosa, Mary L Disis, Vernon C Maino, Holden T Maecker, Cory A Waters

**Affiliations:** 1 BD Biosciences Immunocytometry Systems, 2350 Qume Dr., San Jose, CA 95131, USA; 2 University of Washington, Division of Oncology, 815 Mercer St., Seattle, WA 98109, USA

## Abstract

**Background:**

Monocyte-derived-dendritic-cells (MDDC) are the major DC type used in vaccine-based clinical studies for a variety of cancers. In order to assess whether *in vitro *differentiated MDDC from cryopreserved PBMC of cancer patients are functionally distinct from those of healthy donors, we compared these cells for their expression of co-stimulatory and functional markers. In addition, the effect of cryopreservation of PBMC precursors on the quality of MDDC was also evaluated using samples from healthy donors.

**Methods:**

Using flow cytometry, we compared normal donors and cancer patients MDDC grown in the presence of GM-CSF+IL-4 (immature MDDC), and GM-CSF+IL-4+TNFα+IL-1β+IL-6+PGE-2 (mature MDDC) for (a) surface phenotype such as CD209, CD83 and CD86, (b) intracellular functional markers such as IL-12 and cyclooxygenase-2 (COX-2), (c) ability to secrete IL-8 and IL-12, and (d) ability to stimulate allogeneic and antigen-specific autologous T cells.

**Results:**

Cryopreservation of precursors did affect MDDC marker expression, however, only two markers, CD86 and COX-2, were significantly affected. Mature MDDC from healthy donors and cancer patients up-regulated the expression of CD83, CD86, frequencies of IL-12^+ ^and COX-2^+ ^cells, and secretion of IL-8; and down-regulated CD209 expression relative to their immature counterparts. Compared to healthy donors, mature MDDC generated from cancer patients were equivalent in the expression of nearly all the markers studied and importantly, were equivalent in their ability to stimulate allogeneic and antigen-specific T cells *in vitro*.

**Conclusion:**

Our data show that cryopreservation of DC precursors does not significantly affect the majority of the MDDC markers, although the trends are towards reduced expression of co-stimulatory makers and cytokines. In addition, monocytes from cryopreserved PBMC of cancer patients can be fully differentiated into mature DC with phenotype and function equivalent to those derived from healthy donors.

## Background

Dendritic cells (DC) are promising vehicles for immunotherapy because they are efficient in capturing, processing, and presenting antigens to both naive and memory CD4 and CD8 T cells [1]. To induce strong, antigen-specific T cell responses, DC must mature and express high levels of MHC-antigen complexes and co-stimulatory molecules that enhance interactions with T cells. As a therapeutic modality, the low frequency of DC makes it difficult to readily utilize their unique properties to facilitate innate as well as adaptive immunity. In recent years, major advances have been made in the identification of DC precursors and methods to expand and manipulate these cells *ex vivo*. Thus, significant efforts have been made to utilize cultured DC pulsed with tumor antigens (DC vaccines) to induce anti-tumoral immunity [2-4]. The studies performed to evaluate whether autologous DC precursors from cancer patients are functionally equivalent to those from healthy donors report a defective, semi-differentiated, or intermediate mature phenotype of DC derived from fresh PBMC of cancer patients [5-7]. Furthermore, there are several reports indicating that the cryopreservation of MDDC does not interfere with their activity when compared to freshly derived MDDC from healthy donors as well as cancer patients [8-10]. Although for therapeutic use, generation of DC from cryopreserved PBMC would appear to be an efficient source of precursors, there are very few reports studying the effect of cryopreservation of PBMC precursors on the phenotype and function of MDDC[11,12]. To test the hypothesis that the phenotypic and functional characteristics of MDDC derived from cryopreserved PBMC of cancer patients are different from those derived from healthy donors, we evaluated qualitative and quantitative differences between DC generated from both sources. In addition, the effect of cryopreservation of precursors on the characteristics of MDDC was also evaluated. Specifically, using flow cytometry-based assays, we compared the surface expression of DC-SIGN (CD209), CD83, CD86, and HLA-DR, intracellular expression of IL-12 and COX-2, secretion of inflammatory cytokines, and proliferation of allogeneic and antigen-specific autologous T cells stimulated *in vitro *by DC.

Defective antigen-presenting-cell (APC) function may be associated with impaired HLA expression and lack of co-stimulatory molecules. This is perceived to be one of the primary mechanisms by which tumors evade immune surveillance[7,13,14]. CD83, CD86 and HLA-DR are maturation and co-stimulatory markers expressed on the surface of mature DC activated by various stimuli [15,16]. Up-regulation of HLA-DR and CD86 enable DC to interact more efficiently with T cells and stimulate immune responses. Conversely, the C-type lectin, DC-SIGN (CD209), which is widely recognized as a myeloid DC-specific marker, is down-regulated on DC as a result of maturation [17,18]. The cytokine repertoire of DC matured in the presence of inflammatory stimuli comprises pro-inflammatory cytokines and chemokines, including the T cell inhibitory cytokine IL-10, the Th-1 promoting cytokine IL-12, as well as TNF-α and IL-8 [19-23]. In addition, cyclooxygenase-2 (COX-2), an enzyme responsible for converting arachidonic acid to prostaglandin-E2 (PGE-2), is induced in response to inflammatory stimuli and results in the production of immunosuppressive and pro-inflammatory prostanoids [24-27]. Ability to produce COX-2 can be used as a functional marker of inflammation.

In the present report, MDDC were cultured from fresh and cryopreserved PBMC of healthy donors and cryopreserved PBMC of cancer patients. A comparison of mature MDDC derived from cryopreserved PBMC of the cancer patients and healthy donors revealed that MDDC from cancer patients manifested equivalent levels of expression of virtually all the biomarkers studied including their ability to stimulate T cells.

## Methods

### Donor characteristics

Blood samples from all the donors used in this study were collected after obtaining IRB approvals and appropriate informed consent. Leukapheresis of 16 cancer patients and 11 healthy donors was approved by the IRB of University of Washington (Seattle, WA) and Duke University Medical Center (Durham, NC); PBMC from these samples were prepared using Ficoll-hypaque (Sigma, St. Louis, MO) density gradient separation of leukapheresis products, and processed for cryopreservation [28]. The cancer patient cohort consisted of subjects with advanced cancers of breast, colon, and lung (Table 1). The median age of cancer patients (12 females and 4 males) was 56.5 ± 8.5 yrs. and the median age of the 8 female and 3 male healthy donors was 26 ± 4.5 yrs. For studies with fresh PBMC, blood was collected from 11 in-house healthy donors (3 females and 8 males) in Vacutainer^® ^CPT™ (Cell Preparation Tubes, BD Vacutainer, Franklin Lakes, NJ). The median age of the healthy donors (fresh) was 45 ± 7 yrs. The study was performed retrospectively. Therefore, fresh and cryopreserved samples from the same healthy donors or cancer patients were not available for direct comparison. Neither of the healthy donor control groups was specifically intended to be age or gender-matched with the patient group. Although MDDC were generated from all 16 patients, because of the limited yields, samples from all the patients were not used for evaluation in all the assays.

**Table 1:** 

**Patient ID**	**Sex**	**Type of Cancer/stage**
PH6272	F	Breast/3b
JLN2159	F	Breast/4
DMC6393	F	Breast/3a
94	F	Breast/2
87	F	Breast/3b
72	F	Breast/1
73	F	Breast
74	F	Breast/2
A	M	Colon/4
B	M	Colon/4
C	M	Colon/4
D	M	Colon/4
E	F	Colon/4
F	F	Small bowel/4
G	F	Non small cell lung (NSCLC)
BJH0761	F	Lung

### Generation of MDDC cultures

MDDC were generated as described previously [29] with some modifications. In brief, PBMC were adhered to Petri dishes (BD Falcon, Bedford, MA) for 60 min at 37°C, and the adherent cells were cultured in complete medium [RPMI 1640 (Sigma) supplemented with 1% heat-inactivated plasma, and containing rh-GM-CSF (1000 units/ml, R&D Systems, Minneapolis, MN) and rh-IL-4 (800 units/ml, R&D Systems)]. Cultures were fed with complete medium every other day. On day five, the cultures were split into 6-well plates. On day six, a maturation cocktail consisting of rh-TNF-α, rh-IL-1β, rh-IL-6 (each at 10 ng/mL, R&D Systems), and PGE-2 (1 μg/mL, Sigma) in complete medium was added to half the wells (mature MDDC); the cells from the remaining wells received complete medium alone (immature MDDC). Twenty-four hours later, the non-adherent cells from each group were collected and used for analysis. The culture supernatants were stored at -80°C for assessment of secreted cytokines.

### Surface staining of MDDC for phenotypic analysis

Immature and mature MDDC were stained with CD14- or HLA-DR-FITC, CD86-PE, CD209-PerCP-Cy5.5, and CD83-APC (BD Biosciences, San Jose, CA) for 30 minutes in dark at room temperature. The cells were then washed with PBS containing 1% BSA and 0.1% sodium-azide (wash buffer), fixed in 1% paraformaldehyde, and stored at 4°C in the dark. The samples were analyzed on a FACSCalibur™ flow cytometer (BD Biosciences) within 24 h.

### Detection of intracellular IL-12 and COX-2 by flow cytometry

MDDC collected from day 7 cultures were stimulated in the presence of a secretion inhibitor, brefeldin-A (BFA, 5 μg/mL, Sigma) for 18–20 h in 96-well polypropylene V-bottom plates (BD Falcon) without or with LPS (100 ng/mL, Sigma), or with rh-IFN-γ (1000 U/mL, R&D Systems) + LPS. Cells were washed and surface stained with CD209-PerCP-Cy5.5 and CD14-FITC (BD Biosciences), followed by fixation and permeabilization (Cytofix/Cytoperm solution, BD Biosciences, San Diego, CA). The cells were then stained with PE or APC conjugated anti-IL-12 and PE conjugated anti-COX-2 mAbs (BD Biosciences). The washed and fixed samples were stored at 4°C in the dark and analyzed on a FACSCalibur flow cytometer within 24 h.

### Detection of secreted cytokines by Cytometric Bead Array (CBA)

For detection of secreted cytokines, supernatants from immature and mature MDDC cultures were thawed and analyzed with the Human Inflammation CBA kit (BD Biosciences, San Diego, CA) according to the manufacturer's instructions. Cytokines that had been added to the cultures for maturation (GM-CSF, IL-1β, IL-6, and TNF-α) were excluded from further analysis.

### Allogeneic and antigen-specific autologous T cell stimulation

MLR were performed to test the ability of DC to stimulate allogeneic T cells. PBMC from fresh blood of healthy donors were labeled with 5 μM final concentration of CFSE (Vybrant CFDA-SE Cell Tracer Kit, Molecular Probes, Eugene, OR) for 15 minutes at 37°C. Labeled cells were washed according to manufacturer's instructions and used as responder cells. Mature MDDC from healthy donors and cancer patients were plated at 1 to 2 × 10^5 ^cells/well in a 24-well plate (BD Falcon) in RPMI with 10% heat-inactivated FBS. CFSE-labeled responder PBMC were added to the wells containing MDDC at DC:PBMC ratios of 1:1, 1:5, and 1:20, and the cells were cultured for four days. On day 4, cells were washed and surface stained with CD3-PE, CD209 PerCP-Cy5.5, and CD4-APC (BD Biosciences) as described above. Proliferation was measured as percentage of CD3^+^CD4^+ ^and CD3^+^CD4^- ^(from here on referred to as CD8^+^) cells, excluding the CD209^+ ^MDDC (stimulator cells), with decreased CFSE staining intensity resulting from dilution during cell division (viz., the fluorescence intensity of membrane staining halves with each cell division). Background proliferation of allogeneic responder PBMC in the absence of MDDC stimulators was subtracted for data analysis.

Ability of MDDC to enhance superantigen-specific, recall antigen-specific, and tumor antigen-specific autologous T cell stimulation was respectively measured by using SEB (0.25 μg/ml, List Biological Laboratories, Inc., Campbell, CA), and overlapping peptide mixes of CMV-pp65 (recall antigen), HER2/neu (intracellular domain), MAGE-3, or CEA (commonly expressed tumor antigens) as antigenic stimuli. SEB, a superantigen, was used as generic positive control antigen because the serological status of the donors for any of the commonly-used recall antigens was not known. However, 50%–80% of the adult population in US is CMV-seropositive[30], suggesting that responses might be expected in approximately 50%–80% of the subjects surveyed. Similarly, the most commonly-expressed tumor antigens, e.g., Her-2/neu, MAGE-3 and CEA were selected to evaluate the ability MDDC to stimulate tumor-antigen-specific T cells [31-39]. Mixtures of peptides consisting of 15 amino acid residues, overlapping by 11 amino acids each, were designed to span the sequences of CMV pp65, CEA, MAGE-3, and the intracellular domain (ICD) of HER-2/neu. Sequences were accessed from Genbank [40,41]. All peptide mixes were obtained from SynPep (Dublin, CA) and were reconstituted at 100× concentration in dimethylsulfoxide (DMSO), diluted in PBS and used at 5 μg/ml/peptide (BD Biosciences). A suboptimal concentration of SEB was used to enable the detection of DC-mediated increase in proliferation. Fresh autologous PBMC or thawed and overnight rested autologous PBMC, were labeled with CFSE as described above and used as responder cells to measure antigen-specific proliferation. One to 2 × 10^5 ^MDDC were pulsed with each of the antigens (when sufficient cells were available) for 2 h at 37°C. CFSE-labeled autologous PBMC were added to the wells containing antigen-pulsed MDDC at a DC:PBMC ratio of 1:5. PBMC stimulated with these antigens in the absence of pulsed MDDC served as controls. Cultures were incubated for four days and processed as described above for MLR. Background proliferation of autologous responder PBMC in the absence of any stimulus was subtracted for data analysis

### Statistical analysis

Data were analyzed using Wilcoxon matched pair test (paired-nonparametric: e.g., unstimulated versus stimulated, SEB-stimulated versus DC+SEB-stimulated), and Mann-Whitney test (unpaired-nonparametric: e.g., fresh versus cryopreserved, healthy versus cancer, and immature versus mature). Comparisons of yield, morphology, phenotype, and function were made between fresh PBMC-derived and cryopreserved PBMC-derived MDDC of healthy donors, and between cryopreserved PBMC-derived MDDC of healthy donors and cancer patients. GraphPad Prism statistical software (GraphPad Software Version 4.01, San Diego, CA) was used for data analysis and graphs.

## Results

### Cryopreservation of DC precursors does not significantly affect the majority of the MDDC characteristics

The effect of cryopreservation on the differentiation of DC was studied by comparing the phenotypic and functional properties of mature MDDC derived from cryopreserved PBMC of healthy donors to those from fresh PBMC of healthy donors. Because PBMC from cancer patients were only available in a cryopreserved format, these cells were not available for use in this comparison.

**Figure 1:**
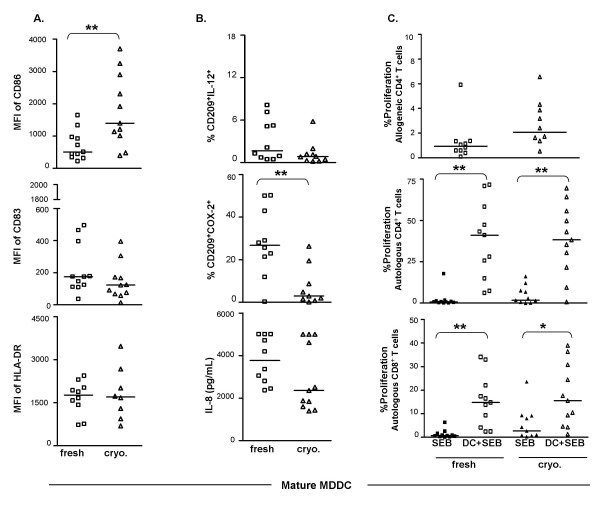
**Comparison of mature MDDC derived from fresh PBMC vs. cryopreserved PBMC of healthy donors**. **A**. Surface phenotype: Mature MDDC derived from fresh or cryopreserved PBMC were stained with antibodies to CD209, CD86, CD83, and HLA-DR as described in Methods. For flow cytometric analysis, a gate was set on the cells with large scatter (size) that were expressing the myeloid DC specific marker CD209. The staining intensities (mean fluorescence intensity, MFI) of CD86, CD83, and HLA-DR were compared between mature MDDC derived from fresh or cryopreserved PBMC. **B**. Functional markers: Mature MDDC derived from fresh or cryopreserved PBMC were cultured for additional 18–20 h in presence of secretion inhibitor BFA. As described in Methods, cells were surface stained with antibodies to CD209, CD14, or CD86, and stained with antibodies to IL-12 and COX-2 for intracellular detection. For flow cytometric analysis, a gate was set on the large cells that also expressed CD209. Results are expressed as percentage of CD209^+ ^cells that were positive for IL-12 (%CD209^+^IL-12^+^) or COX-2 (%CD209^+^COX-2^+^). Amounts of IL-8 (pg/ml) secreted by mature MDDC from each group were detected by using Cytometric Bead Array (CBA) technology (see Methods). Reported quantities (pg/ml) of the cytokines and chemokines reflect the production by 5 × 10^5 ^cells cultured in 3.75 ml medium. **C**. T cell stimulation: Scatter plot in the top panel shows proliferation of allogeneic CD4^+ ^T cells using mature MDDC from fresh and cryopreserved PBMC of healthy donors. One to 2 × 10^5 ^MDDC were mixed with CFSE-labeled allogeneic fresh PBMC at a DC:PBMC ratio of 1:5 in a total volume of 1 ml/well of a 24-well plate. The lower two scatter plots demonstrate enhancement of MDDC mediated SEB-specific autologous CD4^+ ^and CD8^+ ^T cell proliferation. CFSE-labeled autologous PBMC from either fresh or cryopreserved healthy donors were added to the wells containing SEB alone or SEB-pulsed respective autologous mature MDDC at a DC:PBMC ratio of 1:5 as described in Methods. After four days of culture, cells were surface stained with CD3 PE, CD209 PerCP-Cy5.5 and CD4 APC and acquired on a flow cytometer. CD3^+^CD4^+ ^lymphocytes were gated including the blasts and excluding CD209^+ ^MDDC. The percentage of cells showing decreased CFSE staining intensity was reported as %proliferation. Bars in all the scatter plots represent medians. *, statistically significant differences (P < 0.05); **, statistically significant differences (P < 0.01).

Cryopreservation did not significantly affect levels of cell surface expression of CD209 (data not shown), CD83, and HLA-DR (Fig. 1A), or secretion of IL-8 (Fig. 1B). However, CD86 expression was significantly higher on mature MDDC derived from cryopreserved versus fresh PBMC (Fig. 1A).

When intracellular expression of IL-12 was evaluated in mature MDDC from fresh and cryopreserved PBMC, no differences were observed in the frequency of IL-12^+ ^cells in unstimulated (constitutive expression) and LPS-stimulated cultures. Unlike IL-12, cryopreservation of PBMC decreased the frequency of COX-2^+ ^cells in unstimulated mature MDDC cultures (Fig. 1B). In addition, significant increases in COX-2^+ ^cells were observed in LPS and IFN-γ+LPS stimulated mature MDDC from cryopreserved PBMC, compared to the mature MDDC from fresh PBMC (p < 0.03, data not shown).

The ability of mature MDDC derived from fresh and cryopreserved PBMC to stimulate allogeneic T cells was assessed by performing MLR. Mature MDDC prepared from cryopreserved PBMC were not significantly different compared to those from fresh PBMC in stimulating allogeneic CD4^+ ^(p = 0.063, Fig. 1C, Top panel) and CD8^+ ^(p = 0.3527, data not shown) T cell proliferation.

When tested for antigen-specific autologous T cell stimulatory capacity, mature MDDC derived from both fresh PBMC as well as cryopreserved PBMC were able to significantly enhance SEB-specific autologous CD4^+ ^and CD8^+ ^T cell proliferation compared to the stimulation of PBMC with SEB alone (Fig. 1C, middle and bottom graphs). Autologous CD4^+ ^and CD8^+ ^T cell stimulation in response to CMV-pp65, HER2/neu, and MAGE was also higher in the presence of MDDC from the cryopreserved healthy group compared to the stimulation of PBMC with these antigens alone. However, when DC were derived from fresh PBMC, the antigen-specific, DC-driven responses were comparable to those achieved with antigen alone (data not shown). This difference appears to be the result of diminished antigen-specific baseline responses, potentially associated with compromised APC function in cryopreserved PBMC. Addition of antigen-pulsed MDDC to these cultures appears to increase the baseline responses.

When efficiency of autologous T cell stimulation was compared between fresh PBMC-derived and cryopreserved PBMC-derived MDDC, there were no statistically significant differences between antigen-specific (SEB, CMV-pp65, MAGE) CD4^+ ^T cell proliferation (e.g., DC+SEB columns of fresh vs. cryo. in the middle graph in Fig. 1C), with the exception of HER2/neu and CEA where responses of fresh PBMC-derived samples were higher (p < 0.05) compared to the cryopreserved samples (data not shown). There were no significant differences between any of the antigen-specific responses of CD8^+ ^T cells stimulated by these two different groups of MDDC (e.g., the DC+SEB columns of fresh vs. cryo. in bottom graph in Fig. 1C).

### Monocytes from cryopreserved PBMC of cancer patients can differentiate into mature DC

To examine whether the source of precursors (i.e., fresh healthy PBMC, cryopreserved healthy PBMC, or cryopreserved cancer PBMC) affected the maturation-induced changes of MDDC, immature and mature MDDC within each of the three groups were evaluated for their expression of surface and other functional markers.

**Figure 2:**
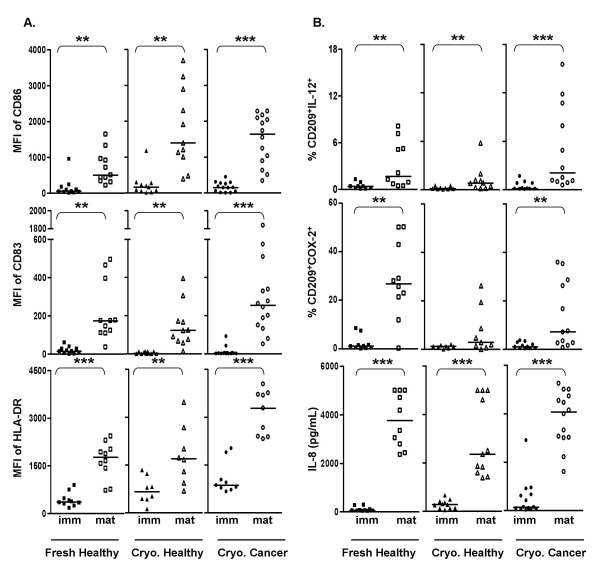
**Effect of maturation on MDDC derived from fresh PBMC of healthy donors (Fresh Healthy), cryopreserved PBMC of healthy donors (Cryo. Healthy), and cryopreserved PBMC of cancer patients (Cryo. Cancer)**. **A**. Surface phenotype: Immature and mature MDDC from each of the three groups were compared for their expression levels (MFI) of CD86, CD83, and HLA-DR. **B**. Function: Immature and mature MDDC were cultured for additional 18–20 h in presence of BFA. Cells were processed and analyzed to evaluate the expression of intracellular IL-12 (% CD209^+^IL-12^+^) or COX-2 (% CD209^+^COX-2^+^). Quantities of secreted IL-8 by immature and mature MDDC from each of these two groups were detected by CBA assay of the culture supernatants collected on day 7. Bars in all the scatter plots represent medians. **, statistically significant differences (P < 0.01); ***, statistically significant differences (P < 0.001).

Compared to immature MDDC, a population of mature MDDC with significantly down-modulated CD209 expression (p < 0.01, not shown), and significantly up-regulated CD86, CD83, and HLA-DR expression was identified in all of the three groups (Fig. 2A). Mature MDDC from all three groups contained significantly higher frequencies of IL-12^+ ^cells without further re-stimulation, when compared to the respective immature MDDC (Fig. 2B, top panel). As shown in Fig. 2B (middle panel), unstimulated mature MDDC cultures from fresh healthy and cryopreserved cancer groups contained significantly higher numbers of COX-2^+ ^cells compared to the corresponding unstimulated immature MDDC.

**Figure 3:**
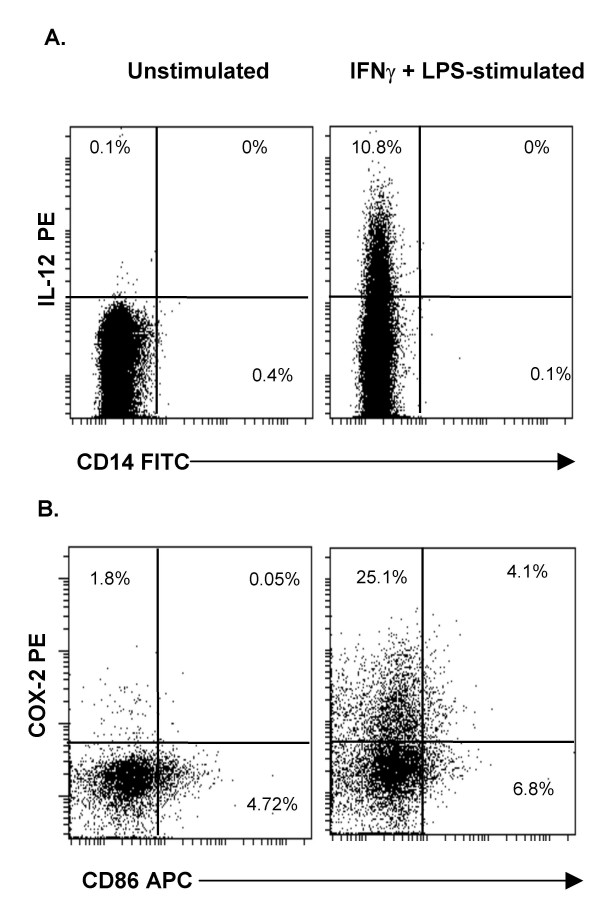
**Intracellular detection of IL-12 and COX-2 in MDDC**. The cells were stimulated (or not) and processed for flow cytometry analysis as described in Methods.**A**. Dot plots in this panel show MDDC, gated on CD209^+^cells that express intracellular IL-12 in unstimulated and IFNγ+LPS-stimulated immature MDDC from fresh PBMC. **B**. Dot plots in this panel show intracellular staining of COX-2 in unstimulated and LPS stimulated immature MDDC from fresh PBMC.

Both immature and mature MDDC from fresh PBMC of healthy donors and cryopreserved PBMC of cancer patients responded to LPS stimulation by displaying a significantly higher frequency of IL-12^+ ^and COX-2^+^cells, compared to the corresponding unstimulated cells (p < 0.05, data not shown). The dot plots in Fig. 3A and 3B show the intracellular staining profiles of IL-12 and COX-2 in unstimulated and IFNγ+LPS-stimulated immature MDDC derived from fresh PBMC.

In all three groups studied, mature MDDC secreted significantly higher amounts of IL-8 compared to the corresponding immature MDDC (Fig. 2B, bottom panel). There were no significant differences in IL-10 and IL-12 secretion when the supernatants from immature MDDC cultures were compared to those from mature MDDC within each group (data not shown).

None of the variables described in the preceding paragraphs of this section, however, correlated with the ability of mature MDDC to stimulate in MLR or antigen-specific autologous T cell stimulation (data not shown).

### Characteristics of mature MDDC from cancer patients are equivalent to those from healthy donors

To determine whether there were differences between the characteristics of MDDC from cancer patients and healthy donors, the phenotypes and functions of these cells were directly compared. Because only cryopreserved PBMC from cancer patients were available, this group was compared to cryopreserved PBMC-derived MDDC from healthy donors.

**Figure 4:**
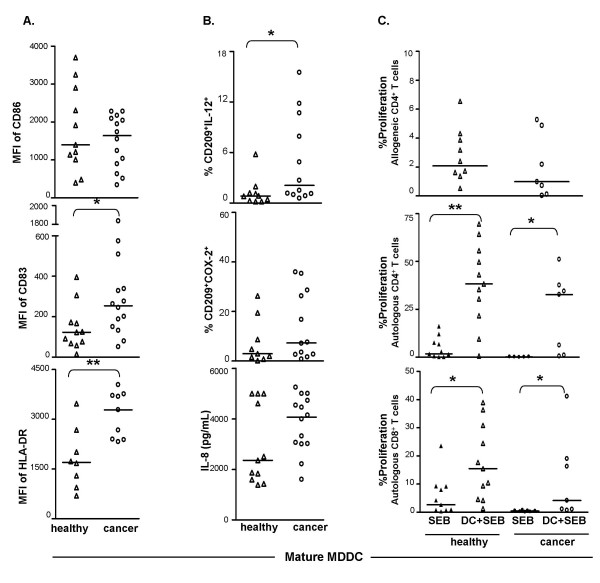
**Comparison of mature MDDC derived from cryopreserved PBMC of healthy donors vs. cancer patients**. **A**. Surface phenotype: Expression levels (MFI) of CD86, CD83, and HLA-DR on mature MDDC derived from healthy donors (healthy) were compared to those derived from cancer patients (cancer). **B**. Function: Mature MDDC from each group were cultured for additional 18–20 h in presence of BFA. Cells were processed and analyzed to evaluate the expression of intracellular IL-12 (%CD209^+^IL-12^+^) or COX-2 (%CD209^+^COX-2^+^) as described earlier. Quantities of secreted IL-8 (pg/ml) by mature MDDC from each of these two groups were detected by CBA assay of the culture supernatants collected on day 7. **C**. T cell stimulation: The top scatter plot shows proliferation of allogeneic CD4^+ ^T cells using mature MDDC from PBMC of healthy donors and cancer patients. The lower two scatter plots demonstrate enhancement of MDDC mediated SEB-specific autologous CD4^+ ^and CD8^+ ^T cell proliferation. Both allogeneic and autologous antigen-specific T cell stimulation assays were set up and percent proliferation was measured as described earlier. Bars in all the scatter plots represent medians. *, statistically significant differences (P < 0.05); **, statistically significant differences (P < 0.01).

There were no significant differences in the expression levels of CD209 (not shown) and CD86 on mature MDDC when cultures derived from cancer patients were compared to cultures from healthy donors (Fig. 4A). Significantly higher expression levels of CD83 and HLA-DR, however, were observed on mature MDDC from cancer patients compared to those from healthy donors (Fig. 4A).

Small but significant increases in IL-12^+ ^cells were observed in mature MDDC derived from the cancer patients as compared to those from healthy donors (Fig. 4B). However, mature MDDC cultures derived from healthy donors and cancer patients contained equivalent frequencies of COX-2^+ ^cells (Fig 4B, middle panel). Mature MDDC from cancer patients as well as from healthy donors up-regulated the frequency of COX-2^+ ^cells in response to LPS (cancer group, p = 0.01; healthy group, p = 0.02) and IFN-γ+LPS stimulation (cancer group, p = 0.02; healthy group, p = 0.004) compared to the respective unstimulated controls (data not shown).

There were no significant differences in IL-8 (Fig. 4B), IL-10, and IL-12 (data not shown) secretion by cryopreserved PBMC-derived MDDC from healthy donors compared to cancer patients.

When tested for the ability to stimulate allogeneic CD4^+ ^T cells (Fig. 4C) and CD8^+ ^T cells (data not shown), mature MDDC prepared from cryopreserved PBMC of cancer patients (five breast cancer and two colon cancer patients) were not significantly different from those of healthy donors.

When the capacity of MDDC to stimulate autologous CD4^+ ^and CD8^+ ^T cell proliferation was tested, all the MDDC preparations derived from both cryopreserved PBMC of healthy donors as well as cancer patients were able to significantly enhance the antigen-specific (i.e., SEB, CMV-pp65, HER2/neu, and MAGE) response compared to stimulation of PBMC with antigens alone. Figure 4C displays data of SEB-specific proliferation of CD4^+ ^(middle graph) and CD8^+ ^(bottom graph) T cells using CFSE-labeled autologous PBMC. MDDC from healthy donors as well as cancer patients stimulated higher CEA-specific CD8^+ ^T cell proliferation compared to stimulation of PBMC with CEA alone.

**Figure 5:**
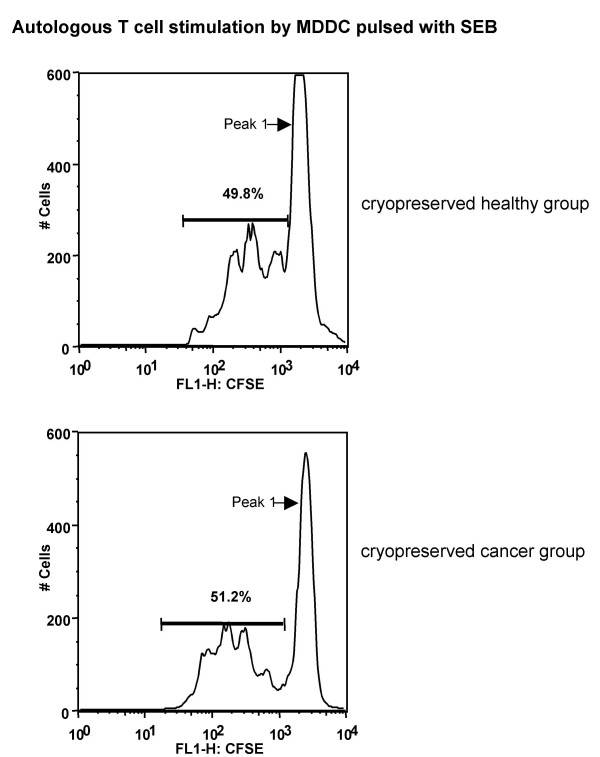
**Enhancement of SEB-specific proliferation of autologous CD4^+ ^T cells by mature MDDC**. Histograms in this figure show the CFSE staining profile of CD4^+ ^T cells from cryopreserved PBMC stimulated with autologous DC pulsed with SEB (A) data from a representative healthy donor, and (**B**) data from a representative cancer patient. Proliferation of CD4^+ ^T cells in presence of SEB alone was 3.1% (healthy donor) and 0.35% (cancer patient). Proliferation is measured as the percentage of cells showing decreased staining intensity of CFSE compared to the intensity of the CFSE^bright ^population (marked as Peak 1 in all histograms). Numbers in all histograms represent %proliferation.

When efficiency of autologous T cell stimulation was compared between these two MDDC groups, there were no statistically significant differences between the antigen-specific (SEB, CMV-pp65, HER2/neu, and MAGE) CD4^+ ^as well as CD8^+ ^T cell proliferation induced by antigen-pulsed MDDC from these two groups (e.g. DC+SEB columns of healthy vs. cancer groups in Fig. 4C). Histograms in Fig. 5 display typical proliferation of CD4^+ ^T cells (dilution of CFSE label) from DC+SEB-stimulated autologous PBMC of a healthy donor and a cancer patient.

## Discussion

Careful manipulation of blood-derived DC precursors using a cocktail of cytokines to generate DC-like cells *in vitro *has been shown to generate efficient antigen-specific T cell immune responses [42]. Advanced understanding of the technologies required to generate human DC, load DC with antigens of interest, and demonstrate a DC-mediated cytotoxic T cell response has enabled the execution of a number of Phase I clinical cancer vaccine trials[43,44]. However, lack of standardization of the source of DC precursors (e.g., fresh vs. cryopreserved), and the type of DC (e.g., immature vs. mature) utilized for therapy make it difficult to compare the outcomes across trials in order to develop better therapeutic strategies[45,46].

In the present report, monocytes were used as precursors to generate DC because they do not require mobilization and can generate enriched populations of DC *in vitro *in 7 days. The effect of cryopreservation on differentiation of precursors into DC-like cells was assessed by performing a cross-sectional comparison of MDDC derived from fresh and cryopreserved PBMC of healthy donors. In addition, cryopreserved PBMC-derived MDDC from cancer patients were compared to cryopreserved PBMC-derived MDDC from healthy donors to evaluate their phenotypic and functional differences. The mature MDDC from cryopreserved PBMC of healthy donors show reduced functional ability compared to the fresh healthy group. However, this observation could also be partially attributed to differences in the donors used in these two groups. The marker expression pattern of mature MDDC from cryopreserved PBMC of cancer patients is at least equivalent to that associated with cryopreserved PBMC of healthy donors.

MDDC generated from all three sources of precursors were morphologically identical, being large in size and having a round or oval nucleus (data not shown). The number of CD209^+ ^MDDC from cryopreserved PBMC of healthy donors was higher, although not significantly, when compared to that of cancer patients (data not shown). However, the immature as well as mature cultures from the cryopreserved healthy donor group contained significantly higher numbers of CD209^+ ^DC compared to the fresh healthy donor group (immature cells, 45.6% [cryopreserved] versus 13.6% [fresh], p < 0.01; mature cells, 53.8% [cryopreserved] versus 24.3% [fresh], p < 0.01). These differences in yields could be due to the effect of cryopreservation, or to blood sample collection by CPT versus leukapheresis, or to differences in donors used for this comparison.

Loss of CD14 expression is a characteristic feature of mature MDDC. MDDC from all the three groups were very low or negative (MFI and percent positive) in their CD14 expression. Consistent with an earlier report, the cytokine/PGE-2 maturation cocktail used in this study provided strong maturation signals for cancer-patient derived DC[15]. In the healthy donor group, CD86 expression was higher on cryopreserved PBMC-derived MDDC compared to those derived from fresh PBMC. This observation suggests that higher expression levels of CD86 on cryopreserved PBMC-derived MDDC could be related to non-specific activation due to components of the freezing medium, such as albumin or DMSO, or the freezing process itself. However, there were no significant differences between CD86 expression on cryopreserved PBMC-derived MDDC from the healthy donors and cancer patients. We also compared the expression of HLA-DR and CD83, both of which are markers of activated and mature DC. MDDC from cancer patients expressed significantly higher levels of HLA-DR and CD83 compared to healthy donors (cryopreserved), confirming their activated and mature phenotype. This increased expression of activation and/or maturation markers on MDDC generated from cryopreserved PBMC of healthy donors and cancer patients is either endogenous condition or could also be due to the uptake of dead cells that may be generated during the freezing/thawing and subsequent culture process.

IL-12 and COX-2 were selected as markers to compare the functional capacity of MDDC. The ability to produce IL-12, which drives the Th1 helper T cell response, is considered to be one of the most important functions of DC because IL-12 secretion appears to correlate with therapeutic efficacy in clinical trials [47-51]. In our study, although there were no significant differences in the frequency of IL-12^+ ^mature MDDC from fresh versus cryopreserved PBMC of healthy donors, culture supernatants from fresh PBMC-derived mature MDDC contained higher levels of secreted IL-12 (range of 5–25 pg/mL/0.5 million cells). The fact that the actual levels of the secreted cytokines were low may be related to the observation that only 23%–54% of the heterogeneous cell population was actually CD209^+ ^DC. The low levels of secreted IL-12 could also be related to the presence of PGE-2 in the maturation cocktail: PGE-2 is a potent inducer of IL-10 and an inhibitor of IL-12 production by APC, including DC. In our study, comparable amounts of IL-10 (median = 11.5 pg/ml) and IL-12 (median = 9.5 pg/ml) were secreted by fresh PBMC-derived mature MDDC from healthy donors. This observation differs from an earlier report showing the absence of IL-12 and presence of IL-10 in cancer patient-derived MDDC culture supernatants[5] and may be associated with differences in the timing of addition of maturation stimuli and harvest of DC culture supernatants. Significantly higher frequencies of IL-12^+ ^cells were observed in mature MDDC cultures derived from cryopreserved PBMC of cancer patients when compared to those from cryopreserved PBMC of healthy donors. However, actual IL-12 secretion by mature MDDC from these two groups was below the limit of detection (<5 pg/ml). This suggests that despite the use of cryopreserved PBMC as a precursor source and the use of PGE-2 for maturation, MDDC from cancer patients could nonetheless still produce intracellular IL-12.

COX-2 is over-expressed in a variety of pre-malignant and malignant conditions. In spite of the demonstrated association of COX-2 with immuno-modulation of APC function in cancer, there are no reports comparing COX-2 expression in DC from healthy donors to those from cancer patients. Other studies have used mRNA expression, immunohistochemistry, or western blot to detect COX-2 in various cells including DC [52-54]. Here, we report the use of flow cytometry to detect COX-2 expressing DC in response to inflammatory stimulation. Mature MDDC from cryopreserved PBMC of healthy donors contained significantly lower numbers of COX-2^+ ^cells compared to those derived from fresh PBMC, indicating that cryopreservation of precursors may adversely affect some functionality of mature MDDC. Mature MDDC derived from cryopreserved PBMC of cancer patients, conversely, showed a trend towards higher numbers of COX-2^+ ^cells compared to those derived from cryopreserved PBMC of healthy donors, suggesting a more activated or inflamed phenotype of cells from cancer patients. These cells may be producing PGE-2 endogenously and thereby regulating DC function, i.e., maturation and IL-12 production *in vivo *[24,55]. It is of interest to note that when LPS-stimulated MDDC were simultaneously stained for intracellular expression of IL-12 and COX-2, about 50–80% of IL-12^+ ^cells also expressed COX-2. Higher frequency of COX-2^+ ^cells and lower amounts of IL-12 production by MDDC matured in presence of PGE-2 may warrant further studies to evaluate whether PGE-2 could be eliminated from maturation cocktail.

Phenotypic and functional deficiencies and decreased *in vitro *T cell stimulatory capacity of DC from patients with chronic myeloid leukemia and breast cancer have been reported [56, 57]. However, it is evident from our data that the expression of co-stimulatory molecules and intracellular functional markers relevant for T cell interaction and activation are largely preserved in MDDC from cancer patients. Consistent with these observations, MDDC in our study were also able to stimulate both allogeneic and antigen-specific autologous T cells. Our autologous T cell stimulation results are in agreement with those reported earlier for advanced breast cancer patients[5] and pancreatic carcinoma patients[12] but different from those described for patients with operable or early stage breast cancer [7, 14, 57]. The differences in these reports could be related to the disease stage or the techniques used in culturing the DC or measuring the response.

It is of interest that MDDC from healthy donors in our study stimulated responses to several cancer antigens. Fresh PBMC-derived DC-driven CD4^+ ^T cell proliferation in response to Her2/neu and CEA was significantly higher compared to that driven by cryopreserved PBMC-derived DC. Whereas there were no differences in the DC-driven CD4^+ ^T cell proliferative responses of these two groups to SEB, pp65 and MAGE antigens. These results suggest that healthy donors are able to make T cell responses to certain cancer antigens, and some of these antigen-specific responses are sensitive to cryopreservation. Cancer-antigen-specific intracellular cytokine expression in T cells has also been observed in a fresh PBMC healthy donor cohort (M. Inokuma, manuscript in preparation). Not surprisingly, the median T cell responses to DC pulsed with cancer antigens were higher in cancer patients compared to those from healthy donors, although the difference was not statistically significant. All of these observations indicate that although cryopreservation affects some functional responses in healthy donors, which could be partially attributed to differences in the donor pool, MDDC from cancer patients are at least as functionally equivalent as those from healthy donors. It is important to note that although the cancer patient cohort used in this study consisted of breast, colon, and lung cancers, the characteristics of the MDDC did not appear to segregate based on the type of cancer. Thus, for example, MDDC from breast cancer patients behaved similarly to those from colon cancer patients. However, a larger number of patients may be required to investigate any cancer-specific differences.

Although altered DC function and differentiation have been proposed as a fundamental mechanism by which tumors evade the immune system, DC from the cancer patients used in the present study appear to possess basic functionality associated with generating efficient T cell responses. The failure of immune surveillance in these patients may more likely be associated with the tumoral environment than with DC functional capacity itself. Thus, tumor-derived immunosuppressive factors, such as vascular endothelial growth factor [58, 59], PGE-2[54], spermine [6], and mechanisms such as apoptosis of DC and T cells [60, 61], Fas/FasL interaction [62], TLR-4 mediated resistance of tumor cells to CTL attack [63], as well as defective maturation of hematopoetic cells [64] may obstruct effective *in vivo *immune responses by inhibiting endogenous DC function. This suggests that the negative influence of endogenously-growing tumors on DC function may be partially responsible for the mixed success of clinical trials reported so far. Increased understanding of tumor-host interactions may help uncover these phenomena and allow better harnessing of the immune system for effective cancer immunotherapy.

## Conclusion

Our data suggest that monocytes from cryopreserved PBMC of cancer patients can be fully differentiated into mature DC with the phenotype and function similar to or better than those derived from healthy donors. The apparent inability of these patients to mount an effective immune response against their tumor antigens seems to be not necessarily related to defective DC phenotype. Furthermore, autologous *in vitro *differentiated DC from cryopreserved PBMC of cancer patients may be a viable option for immunotherapy.

## Competing interests

SAG, SB, JJR, VCM, HTM, CAW are employed by a company whose products and potential products were used in the present work. MLD and CDR have no competing interests.

## Authors' contributions

SAG and CAW designed and supervised the study. SAG, SB and JJR carried out the experiments. CDR prepared and provided cryopreserved PBMC. SAG analyzed the data, and wrote the manuscript with input from HTM, CAW, MLD, and VCM. HTM and CAW contributed equally to the editing of this manuscript. MLD and VCM supported the study. All authors have read and approved the final manuscript.

## References

[B1] Banchereau J, Steinman RM (1998). Dendritic cells and the control of immunity. Nature.

[B2] Fecci PE, Mitchell DA, Archer GE, Morse MA, Lyerly HK, Bigner DD, Sampson JH (2003). The history, evolution, and clinical use of dendritic cell-based immunization strategies in the therapy of brain tumors. J Neurooncol.

[B3] O'Neill DW, Adams S, Bhardwaj N (2004). Manipulating dendritic cell biology for the active immunotherapy of cancer. Blood.

[B4] Palucka AK, Dhodapkar MV, Paczesny S, Burkeholder S, Wittkowski KM, Steinman RM, Fay J, Banchereau J (2003). Single injection of CD34+ progenitor-derived dendritic cell vaccine can lead to induction of T-cell immunity in patients with stage IV melanoma. J Immunother.

[B5] Pedersen AE, Thorn M, Gad M, Walter MR, Johnsen HE, Gaarsdal E, Nikolajsen K, Buus S, Claesson MH, Svane IM (2005). Phenotypic and functional characterization of clinical grade dendritic cells generated from patients with advanced breast cancer for therapeutic vaccination. Scand J Immunol.

[B6] Della Bella S, Gennaro M, Vaccari M, Ferraris C, Nicola S, Riva A, Clerici M, Greco M, Villa ML (2003). Altered maturation of peripheral blood dendritic cells in patients with breast cancer. Br J Cancer.

[B7] Gabrilovich DI, Corak J, Ciernik IF, Kavanaugh D, Carbone DP (1997). Decreased antigen presentation by dendritic cells in patients with breast cancer. Clin Cancer Res.

[B8] Westermann J, Korner IJ, Kopp J, Kurz S, Zenke M, Dorken B, Pezzutto A (2003). Cryopreservation of mature monocyte-derived human dendritic cells for vaccination: influence on phenotype and functional properties. Cancer Immunol Immunother.

[B9] Szmania S, Yi Q, Cottler-Fox M, Rosen NA, Freeman J, Kordsmeier BJ, Moreno A, Shi J, Barlogie B, Tricot G, van Rhee F (2005). Clinical-grade myeloma Ag pre-loaded DC vaccines retain potency after cryopreservation. Cytotherapy.

[B10] John J, Dalgleish A, Melcher A, Pandha H (2005). Cryopreserved dendritic cells for intratumoral immunotherapy do not require re-culture prior to human vaccination. J Immunol Methods.

[B11] Makino M, Baba M (1997). A cryopreservation method of human peripheral blood mononuclear cells for efficient production of dendritic cells. Scand J Immunol.

[B12] Piemonti L, Monti P, Zerbi A, Balzano G, Allavena P, Di Carlo V (2000). Generation and functional characterisation of dendritic cells from patients with pancreatic carcinoma with special regard to clinical applicability. Cancer Immunol Immunother.

[B13] Drexhage HA, Mooy P, Jansen A, Kerrebijn J, Allaerts W, Tas MP (1993). Dendritic cells in tumor growth and endocrine diseases. Adv Exp Med Biol.

[B14] Kichler-Lakomy C, Budinsky AC, Wolfram R, Hellan M, Wiltschke C, Brodowicz T, Viernstein H, Zielinski CC (2006). Deficiences in phenotype expression and function of dentritic cells from patients with early breast cancer. Eur J Med Res.

[B15] Cella M, Engering A, Pinet V, Pieters J, Lanzavecchia A (1997). Inflammatory stimuli induce accumulation of MHC class II complexes on dendritic cells. Nature.

[B16] Whiteside TL, Stanson J, Shurin MR, Ferrone S (2004). Antigen-processing machinery in human dendritic cells: up-regulation by maturation and down-regulation by tumor cells. J Immunol.

[B17] Geijtenbeek TB, Krooshoop DJ, Bleijs DA, van Vliet SJ, van Duijnhoven GC, Grabovsky V, Alon R, Figdor CG, van Kooyk Y (2000). DC-SIGN-ICAM-2 interaction mediates dendritic cell trafficking. Nat Immunol.

[B18] Relloso M, Puig-Kroger A, Pello OM, Rodriguez-Fernandez JL, de la Rosa G, Longo N, Navarro J, Munoz-Fernandez MA, Sanchez-Mateos P, Corbi AL (2002). DC-SIGN (CD209) expression is IL-4 dependent and is negatively regulated by IFN, TGF-beta, and anti-inflammatory agents. J Immunol.

[B19] de Saint-Vis B, Fugier-Vivier I, Massacrier C, Gaillard C, Vanbervliet B, Ait-Yahia S, Banchereau J, Liu YJ, Lebecque S, Caux C (1998). The cytokine profile expressed by human dendritic cells is dependent on cell subtype and mode of activation. J Immunol.

[B20] Czerniecki BJ, Cohen PA, Faries M, Xu S, Roros JG, Bedrosian I (2001). Diverse functional activity of CD83+ monocyte-derived dendritic cells and the implications for cancer vaccines. Crit Rev Immunol.

[B21] Spisek R, Bretaudeau L, Barbieux I, Meflah K, Gregoire M (2001). Standardized generation of fully mature p70 IL-12 secreting monocyte-derived dendritic cells for clinical use. Cancer Immunol Immunother.

[B22] Trinchieri G (1994). Interleukin-12: a cytokine produced by antigen-presenting cells with immunoregulatory functions in the generation of T-helper cells type 1 and cytotoxic lymphocytes. Blood.

[B23] Nagorsen D, Marincola FM, Panelli MC (2004). Cytokine and chemokine expression profiles of maturing dendritic cells using multiprotein platform arrays. Cytokine.

[B24] Fogel-Petrovic M, Long JA, Knight DA, Thompson PJ, Upham JW (2004). Activated human dendritic cells express inducible cyclo-oxygenase and synthesize prostaglandin E2 but not prostaglandin D2. Immunol Cell Biol.

[B25] Ruitenberg JJ, Waters CA (2003). A rapid flow cytometric method for the detection of intracellular cyclooxygenases in human whole blood monocytes and a COX-2 inducible human cell line. J Immunol Methods.

[B26] Sombroek CC, Stam AG, Masterson AJ, Lougheed SM, Schakel MJ, Meijer CJ, Pinedo HM, van den Eertwegh AJ, Scheper RJ, de Gruijl TD (2002). Prostanoids play a major role in the primary tumor-induced inhibition of dendritic cell differentiation. J Immunol.

[B27] Gualde N, Harizi H (2004). Prostanoids and their receptors that modulate dendritic cell-mediated immunity. Immunol Cell Biol.

[B28] Disis ML, dela Rosa C, Goodell V, Kuan LY, Chang JC, Kuus-Reichel K, Clay TM, Kim Lyerly H, Bhatia S, Ghanekar SA, Maino VC, Maecker HT (2006). Maximizing the retention of antigen specific lymphocyte function after cryopreservation. J Immunol Methods.

[B29] Thurner B, Roder C, Dieckmann D, Heuer M, Kruse M, Glaser A, Keikavoussi P, Kampgen E, Bender A, Schuler G (1999). Generation of large numbers of fully mature and stable dendritic cells from leukapheresis products for clinical application. J Immunol Methods.

[B30] Staras SA, Dollard SC, Radford KW, Flanders WD, Pass RF, Cannon MJ (2006). Seroprevalence of cytomegalovirus infection in the United States, 1988-1994. Clin Infect Dis.

[B31] Kern F, Surel IP, Faulhaber N, Frommel C, Schneider-Mergener J, Schonemann C, Reinke P, Volk HD (1999). Target structures of the CD8(+)-T-cell response to human cytomegalovirus: the 72-kilodalton major immediate-early protein revisited. J Virol.

[B32] Maecker HT, Dunn HS, Suni MA, Khatamzas E, Pitcher CJ, Bunde T, Persaud N, Trigona W, Fu TM, Sinclair E, Bredt BM, McCune JM, Maino VC, Kern F, Picker LJ (2001). Use of overlapping peptide mixtures as antigens for cytokine flow cytometry. J Immunol Methods.

[B33] Osugi Y, Vuckovic S, Hart DN (2002). Myeloid blood CD11c(+) dendritic cells and monocyte-derived dendritic cells differ in their ability to stimulate T lymphocytes. Blood.

[B34] Lau R, Wang F, Jeffery G, Marty V, Kuniyoshi J, Bade E, Ryback ME, Weber J (2001). Phase I trial of intravenous peptide-pulsed dendritic cells in patients with metastatic melanoma. J Immunother.

[B35] Schuler G, Schuler-Thurner B, Steinman RM (2003). The use of dendritic cells in cancer immunotherapy. Curr Opin Immunol.

[B36] Figdor CG, de Vries IJ, Lesterhuis WJ, Melief CJ (2004). Dendritic cell immunotherapy: mapping the way. Nat Med.

[B37] Ridgway D (2003). The first 1000 dendritic cell vaccinees. Cancer Invest.

[B38] Dhodapkar MV, Steinman RM, Sapp M, Desai H, Fossella C, Krasovsky J, Donahoe SM, Dunbar PR, Cerundolo V, Nixon DF, Bhardwaj N (1999). Rapid generation of broad T-cell immunity in humans after a single injection of mature dendritic cells. J Clin Invest.

[B39] Jonuleit H, Giesecke-Tuettenberg A, Tuting T, Thurner-Schuler B, Stuge TB, Paragnik L, Kandemir A, Lee PP, Schuler G, Knop J, Enk AH (2001). A comparison of two types of dendritic cell as adjuvants for the induction of melanoma-specific T-cell responses in humans following intranodal injection. Int J Cancer.

[B40] Morse MA, Mosca PJ, Clay TM, Lyerly HK (2002). Dendritic cell maturation in active immunotherapy strategies. Expert Opin Biol Ther.

[B41] Schuler-Thurner B, Schultz ES, Berger TG, Weinlich G, Ebner S, Woerl P, Bender A, Feuerstein B, Fritsch PO, Romani N, Schuler G (2002). Rapid induction of tumor-specific type 1 T helper cells in metastatic melanoma patients by vaccination with mature, cryopreserved, peptide-loaded monocyte-derived dendritic cells. J Exp Med.

[B42] McIlroy D, Gregoire M (2003). Optimizing dendritic cell-based anticancer immunotherapy: maturation state does have clinical impact. Cancer Immunol Immunother.

[B43] Yuan A, Yu CJ, Shun CT, Luh KT, Kuo SH, Lee YC, Yang PC (2005). Total cyclooxygenase-2 mRNA levels correlate with vascular endothelial growth factor mRNA levels, tumor angiogenesis and prognosis in non-small cell lung cancer patients. Int J Cancer.

[B44] Whittaker DS, Bahjat KS, Moldawer LL, Clare-Salzler MJ (2000). Autoregulation of human monocyte-derived dendritic cell maturation and IL-12 production by cyclooxygenase-2-mediated prostanoid production. J Immunol.

[B45] Pockaj BA, Basu GD, Pathangey LB, Gray RJ, Hernandez JL, Gendler SJ, Mukherjee P (2004). Reduced T-cell and dendritic cell function is related to cyclooxygenase-2 overexpression and prostaglandin E2 secretion in patients with breast cancer. Ann Surg Oncol.

[B46] Harizi H, Juzan M, Pitard V, Moreau JF, Gualde N (2002). Cyclooxygenase-2-issued prostaglandin e(2) enhances the production of endogenous IL-10, which down-regulates dendritic cell functions. J Immunol.

[B47] Eisendle K, Lang A, Eibl B, Nachbaur D, Glassl H, Fiegl M, Thaler J, Gastl G (2003). Phenotypic and functional deficiencies of leukaemic dendritic cells from patients with chronic myeloid leukaemia. Br J Haematol.

[B48] Satthaporn S, Robins A, Vassanasiri W, El-Sheemy M, Jibril JA, Clark D, Valerio D, Eremin O (2004). Dendritic cells are dysfunctional in patients with operable breast cancer. Cancer Immunol Immunother.

[B49] Gabrilovich DI, Ishida T, Nadaf S, Ohm JE, Carbone DP (1999). Antibodies to vascular endothelial growth factor enhance the efficacy of cancer immunotherapy by improving endogenous dendritic cell function. Clin Cancer Res.

[B50] Ohm JE, Carbone DP (2002). Immune dysfunction in cancer patients. Oncology (Williston Park).

[B51] Kiertscher SM, Luo J, Dubinett SM, Roth MD (2000). Tumors promote altered maturation and early apoptosis of monocyte-derived dendritic cells. J Immunol.

[B52] Whiteside TL (2002). Apoptosis of immune cells in the tumor microenvironment and peripheral circulation of patients with cancer: implications for immunotherapy. Vaccine.

[B53] Whiteside TL, Rabinowich H (1998). The role of Fas/FasL in immunosuppression induced by human tumors. Cancer Immunol Immunother.

[B54] Huang B, Zhao J, Li H, He KL, Chen Y, Mayer L, Unkeless JC, Xiong H (2005). Toll-like receptors on tumor cells facilitate evasion of immune surveillance. Cancer Res.

[B55] Ishida T, Oyama T, Carbone DP, Gabrilovich DI (1998). Defective function of Langerhans cells in tumor-bearing animals is the result of defective maturation from hemopoietic progenitors. J Immunol.

